# Role of glutamine metabolism in tuberculosis pathogenesis: a mini review

**DOI:** 10.3389/ftubr.2024.1432880

**Published:** 2024-07-10

**Authors:** Sadiya Parveen, William R. Bishai

**Affiliations:** Center for Tuberculosis Research, Department of Medicine, Johns Hopkins School of Medicine, Baltimore, MD, United States

**Keywords:** tuberculosis, host-directed therapies, glutamine metabolism, immunometabolism, infectious diseases

## Abstract

*Mycobacterium tuberculosis* (*Mtb*) has remained one of the major infectious disease killers for generations and generations. In 2023 alone, this ancient disease was responsible for the death of 1.4 million individuals and has infected 10.6 million people. With the ever-evolving multi- and extremely resistant *Mtb* strains, the need for novel and effective drugs requiring shorter treatment regimens represents an urgent medical need for the development of new drugs. Over the last two decades, the field of host-directed therapy as a potential novel avenue for new approaches to TB treatment, either as a mono or adjuvant therapy, has garnered increasing attention. Among many host-directed targets, host immunometabolism has emerged as one of the most attractive targets for developing new host-directed therapies. As one of the most successful bacterial pathogens, *Mtb* has evolved several mechanisms to modulate numerous host metabolic pathways, including glycolysis, glutaminolysis, Kreb cycle, and oxidative phosphorylation. This mini review will focus on glutamine metabolism and its emergence as a potential target for treating tuberculosis (TB). In the last several decades, the role of glutamine metabolism in cancer and neurological disorders has been extensively studied. However, the association of glutamine metabolism with infectious disease has remained underappreciated. The aim of this review is to not only discuss the current knowledge in the field but also the existing knowledge gap that needs further exploration.

## Introduction

*Mycobacterium tuberculosis (Mtb*) is one of the most successful infectious agents worldwide. In the year 2023 alone, *Mtb* infected 10.6 million individuals and killed 1.3 million individuals (1). *Mtb*, the ancient pathogen, is known to modulate several host pathways to aid its own establishment, progression, and dissemination. These host pathways include various metabolic pathways such as glycolysis, pentose phosphate pathway, glutamine metabolism, fatty acid oxidation, Kreb cycle, and oxidative phosphorylation (2–5). Over the past several decades, glutamine metabolism has garnered increasing attention as a therapeutic target in the field of cancer and neurological disorders. However, there is a fundamental gap in the knowledge of how glutamine metabolism contributes to tuberculosis pathogenesis. Over the last few years, several researchers have published data that strongly supports the notion that glutamine metabolism plays a critical role in TB pathogenesis. This review attempts to compile all the studies and present them in the context of TB pathogenesis. The mini-review also discusses the unanswered questions in the field and potential strategies (including pitfalls) to find the answers.

## Glutamine is the most abundant amino acid in the human body

Glutamine is the most abundant amino acid of all 20 amino acids in the human body. The average human plasma contains ~500–800 μM/L glutamine (after 12 h fasting), which is 20% of the total amino acid pool in the plasma (6). In tissues like skeletal muscles and liver, glutamine concentration can account for as much as 40%−80% of the total amino acid pool, further reinforcing the status of glutamine as the most abundant amino acid in the body (7, 8). Despite this heavy abundance, glutamine supplementation remains non-essential under normal physiological conditions. As a versatile amino acid, glutamine participates in a variety of metabolic pathways, such as synthesis of amino acids (e.g., asparagine), purines, pyrimidines, amino sugars, nicotinamide adenine dinucleotide phosphate (NADPH), glutathione, in addition to providing substrates for tricarboxylic acid (TCA) cycle. In short, glutamine serves as a fuel and nitrogen donor via ammonia for the pathways critical for cellular growth, proliferation, differentiation, and maintenance.

## Glutamine transport and its cellular fates

**Figure 1:**
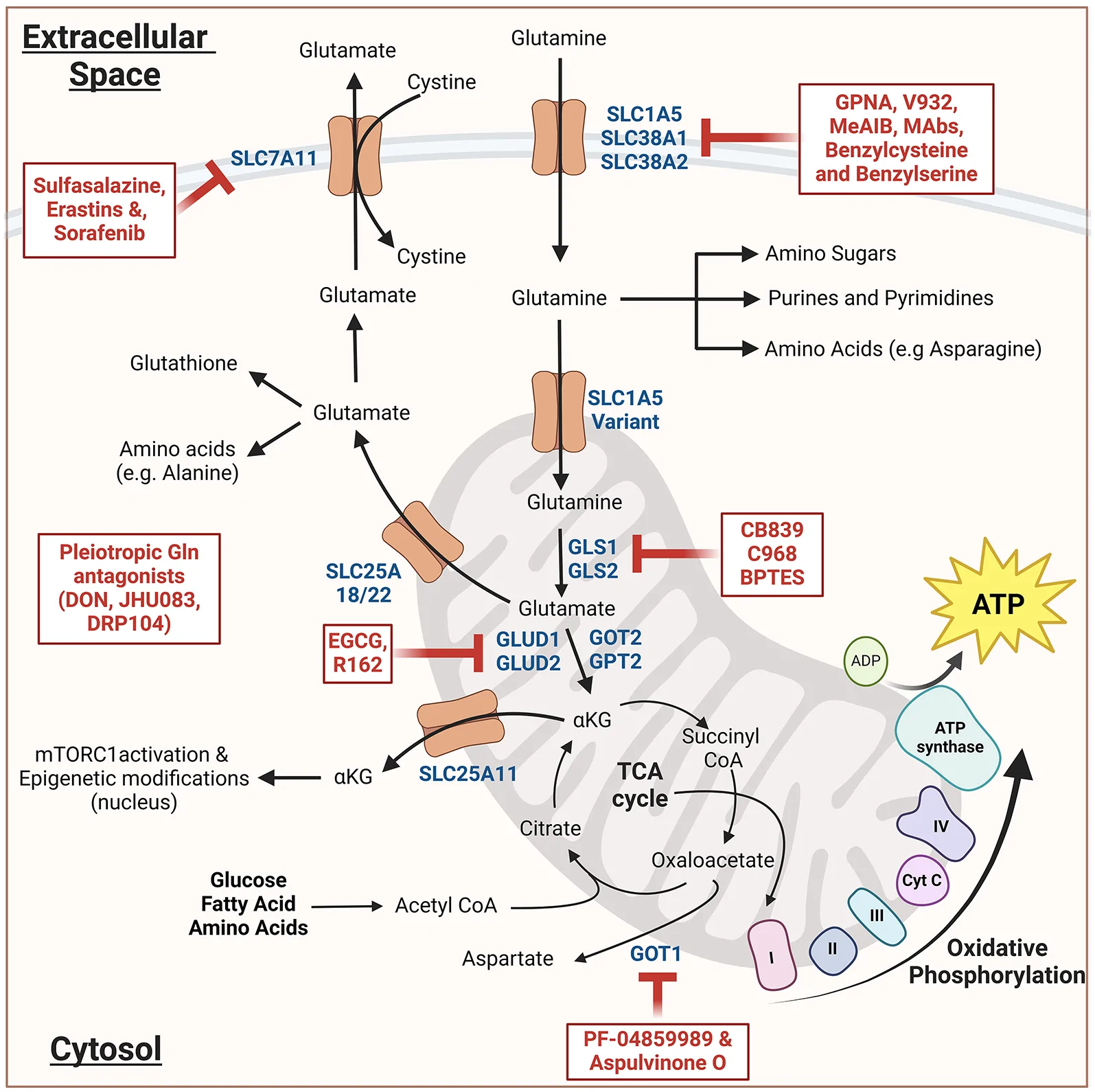
Schematic representation of glutamine metabolism and its inhibitors: Glutamine enters the cytosol and then either engages in various biosynthetic pathways or enters the mitochondria wherein it undergoes glutaminolysis. The resulting alpha-ketoglutarate (αKG) either enters Kreb's cycle leading to oxidative phosphorylation and ATP generation. Alternatively, it can lead to the mTORC1 activation and epigenetic modifications. The resulting glutamate when transported to the cytosol, generates glutathione and non-essential amino acids. All the inhibitors are indicated in red; dark blue indicates the important components of glutamine metabolism. The schematic was created using Biorender.com (Tornonto, Canada) by SP.

As shown in Figure 1, Glutamine enters the cells via one of the several glutamine transporters like SLC1A5 (ASCT2), SLC38A1, or SLC38A2 present in the plasma membrane (9). Once inside the cell, glutamine may either stay in the cytosol and participate in the synthesis of purines, pyrimidines, amino sugars, or non-essential amino acids. Alternatively, glutamine can be transported to the mitochondrial matrix via a mitochondrial glutamine transporter (a SLC1A5 variant), where it undergoes glutaminolysis. First, glutaminases (GS) convert glutamine to glutamate, and then glutamate dehydrogenase (and several other aminotransferases) convert glutamate into alpha-ketoglutarate (ɑ-KG), which then serves as a substrate for the Kreb's cycle, eventually fueling the oxidative phosphorylation and ATP generation. Under hypoxic conditions, SLC25A11-driven transport of ɑ-KG from mitochondria to cytosol plays a critical role in mTROC1 activation and epigenetic modification via ɑ-KG-dependent dioxygenases (10). In addition to ɑ-KG, mitochondria can also export glutamine-derived glutamate to the cytosol via SLC25A18/22, which drives the synthesis of glutathione and non-essential amino acid synthesis in addition to cystine import (11).

## Glutamine distribution in the tissues

Glutamine concentration in various organs and tissues is driven by its synthesis, release, and uptake. Tissues like brain, skeletal muscles, lungs, adipose tissue, and liver exhibit high *de novo* glutamine synthesis activity. Glutamine synthesis is driven by an ATP-dependent glutamine synthetase (GS) that produces glutamine from glutamate and ammonia. *De novo* Glutamine synthesis serves two important functions; first, it provides glutamine required for rapid cell growth and proliferation. Second, it aids pH homeostasis by removing excess ammonia from the cells. Alterations in GLS activity have been associated with various pathologies like cancer, hyperammonemia, and neurological disorders, including behavior abnormalities, as well as cognitive and motor deficits (12). Tissues like intestinal mucosa, kidney, leukocytes, and vascular endothelial cells tend to heavily rely on glutamine consumption by upregulating the expression of glutaminase (GLS), the enzyme that catalyzes the conversion of glutamine to glutamate. There are two distinct GLS isoforms, (1) kidney-type glutaminase (GLS1) and (2) liver-type glutaminase (GLS2), encoded by genes located on chromosome 2 and 12 respectively (13–18). GLS1 has three additional isoforms; GLS or KGA (which corresponds to a longer transcript isoform), GLS C or GAC (which corresponds to a shorter transcript isoform), and GAM (with no catalytic activity). While GLS1 upregulation causes tumor progression in most cancers, the role of GLS2 seems more context specific. While GLS2 acts as a tumor-suppressor gene and prevents metastasis in many cancers (19), its upregulation has been shown to potentiate the occurrence and development of neuroblastoma tumors (20, 21).

## Glutamine as a conditionally essential immunonutrient

Under normal physiological conditions, glutamine remains non-essential as the human body can endogenously produce from 40 to 80 g/L glutamine (22, 23). However, under catabolic conditions such as infections, glutamine becomes conditionally essential. Additionally, by virtue of working under these harsh and nutrient-deprived/restricted conditions, immune cells tend to heavily rely on glutamine as both the fuel and nitrogen source (24, 25). Paradoxically, under such highly catabolic conditions, several tissues increase their glutamine consumption while skeletal muscles tend to decrease their contribution to the serum glutamine concentration, further limiting glutamine availability for the immune cells. Such glutamine-depleted conditions often diminish the ability of the immune system to optimally combat the pathological agent/condition and lead to the worsening of the pathology. Hence, glutamine has long been considered a critical nutrient for the immune system, “an immunonutrient.” Depending upon the pathological condition, glutamine supplementation has been shown to improve clinical outcomes and prevent life-threatening conditions (26, 27). Almost all immune cells, including T-cells, macrophages, B-cells, and neutrophils, depend on glutamine uptake and utilization for their proliferation, differentiation, and activation (28). This heavy reliance of the immune system upon glutamine is a critical immune vulnerability. It is possible that pathogens may have evolved mechanisms to exploit this vulnerability to promote their own replication and dissemination.

## Glutamine as one of the drivers of immunosuppression

During pathological conditions such as cancer and infections, the immune cells driving immunosuppression include myeloid-derived suppressor cells (MDSCs), regulatory T-cells (Tregs), and M2 macrophages (29–32). These immunosuppressive cells create an artificial scarcity of glutamine by ramping up the import and utilization of this amino acid. The artificial scarcity of glutamine hampers T-cells' ability to proliferate, activate, and differentiate into helper T-cells. Glutamine-deprived T-cells have also been shown to differentiate into FoxP3+ Tregs instead of Th1 helper cells, a defect that could be circumvented by simply feeding cells the cell-permeable ɑ-KG (33). L-glutamine also fuels the maturation process of MDSCs and is essential for their immunosuppressive activity (34). Additionally, glutamine favors the activation of M2 macrophages through the glutamine–UDP-*N*-acetylglucosamine pathway and glutamine-derived ɑ-KG (28, 35). Additionally, B-cells, especially the IL-10 expressing regulatory B-cells, which are critical for immune tolerance, have been shown to upregulate glutaminolysis (36, 37). Accordingly, in murine cancer models, blockade of glutamine metabolism has been shown to deplete MDSCs and Tregs populations and to metabolically reprogram M2 macrophages, alleviating immunosuppression and enhancing anti-tumor T-cell immunity (38–40).

## Glutamine metabolism in infectious diseases (bacterial, viral, and fungal)

The speculation that glutamine plays a substantial role in infectious diseases dates back to 1975 when depletion of skeletal muscle Glutamine pool was detected under stress conditions like surgery, trauma, and inflammatory conditions. Several studies have shown that glutamine supplementation improved clinical outcomes and decreased mortality of severely ill patients with sepsis. In prolonged abdominal sepsis patients (*n* = 14), muscle glutamine concentration was identified as the single most reliable factor that effectively discriminated survivors from non-survivors (6, 8). The survivors exhibited higher glutamine and lower branched-chain amino acid levels in their muscles. In another study, low plasma glutamine concentration in critically ill patients was directly correlated with higher mortality rates (41). However, this initial enthusiasm was modestly dampened by the studies that found no correlation between plasma glutamine concentration and favorable outcomes in critically ill patients (41, 42). Despite these contradictory findings, several studies have observed substantial derangements in the glutamine distribution, transport, and synthesis in the tissues of patients carrying severe infections (43). During infections, skeletal muscles release two-fold as much glutamine, indicating a substantial increase in the *de novo* GS-mediated glutamine synthesis.

Alterations in glutamine metabolism have also been observed in several bacterial, viral, and fungal infections, including Human Immunodeficiency Virus (HIV), *Mycobacterium tuberculosis* (*Mtb*), COVID-19 and *Aspergillus fumigatus*. In 2017, Djoko et al. (44) demonstrated that *Escherichia coli* alters its glutamine metabolism and may utilize glutamine to overcome host-imposed metal toxicity. More recently, Turner et al. (45) showed that glutamine supplementation can protect host tissues from cholesterol-dependent cytotoxin secreted by pathogenic bacteria. HIV infection elevates glutamine levels and alters several key glutamine metabolism enzymes in human T-cells (46). In addition, the blockade of Glutamine metabolism was also shown to reverse a cognition deficit in the mouse model of HIV-associated neurocognitive disorders (47). In COVID-19 patients, a decline in circulatory glutamine levels directly correlates with the disease severity (48–54). In another meta-analysis study performed with West African cohorts, high plasma glutamine concentration was associated with a lower risk of COVID-19 infections and disease severity (55). Blocking glutamine metabolism was also reported to check the progression of *Aspergillus fumigatum* in macrophages and in the experimental model of Aspergillosis (56). These studies strongly support that alterations in the host glutamine metabolism may be a unique metabolic signature of several bacterial, viral, and fungal infections.

## Glutamine metabolism alterations as the potential driver of TB pathogenesis

In the majority of patients who present with TB, the lung is the primary organ affected. The lung is also the organ with the second most abundant glutamine levels, with hitherto *de novo* glutamine synthesis activity. Alterations in lung glutamine levels and metabolism have been observed in several critical illnesses. For example, the lungs of septic surgical patients had 850% higher glutamine levels compared to the preoperative controls. Exogenous glutamine supplementation is still used in the Intensive Care Unit to prevent post-operative complications. Glutamine supplementation has also exerted beneficial effects on several respiratory and pulmonary ailments, such as asthma and acute respiratory distress syndrome. However, the impact of exogenous glutamine supplementation on TB progression has not been investigated so far.

Nonetheless, several studies have linked glutamine metabolism to TB pathogenesis. Glutamine is the primary nitrogen donor in *Mtb*-infected macrophages (57). With one exception (58), several studies have identified decreased levels of glutamine in serum/blood as one of the prominent diagnostic markers that distinguish active TB patients from latent TB patients and healthy controls (59–62). *Mtb* infection has also been shown to affect the expression of several genes associated with glutamine metabolism in both human macrophages and TB patients (63). The same study also demonstrated that perturbing glutamine pathway either via glutamine depletion or by using specific chemical inhibitors or due to genetic polymorphism (e.g., GLS2, SLC7A5, and SLC1A5), decreased T-cell associated cytokine production by the macrophages (PMID: 30541099).

Similarly, Vreiling et al. (64) demonstrated substantial alterations in the expression of multiple glutamine metabolism pathway transcripts in *Mtb*-infected M2 macrophages. More recently, Jiang et al. (65) using stable isotope labeling of glutamine and glucose followed by metabolomics, reported that M1 macrophages preferentially utilize glutamine, and the chemical or genetic ablation of glutamine synthase perturbs M1 polarization of the macrophages. Parveen et al. (66, 67) have reported that inhibiting glutamine metabolism in a murine model of TB has dual immunomodulatory and antibacterial activities. The study demonstrated that glutamine metabolism inhibition causes early recruitment of activated T-cells, reduced frequency of immunosuppressive myeloid cells, and enhanced antimycobacterial activity of macrophages concomitant with upregulation of host-protective metabolic pathways. Eventually, glutamine metabolism inhibition was also shown to reduce lung bacillary burden and improve lung histopathology and survival in murine hyperacute models of TB (66, 67).

Overall, these studies demonstrate that *Mtb* infection alters glutamine metabolism in infected macrophages, mice, and TB patients, and hence modulation of the glutamine levels and/or metabolism has the potential as an effective host-directed therapy.

## Glutamine supplementation as an adjunctive therapy for TB

Several studies performed with patient cohorts from Africa, Indonesia, and Georgia have identified lower circulatory glutamine levels as a potential diagnostic signature of TB patients. The lower glutamine levels could be due to either decreased GLS-mediated synthesis or increased glutamine utilization/uptake. Further studies need to be performed to dissect the mechanism driving the lower glutamine levels in TB patients. Regardless of the mechanism, however, glutamine supplementation to restore circulatory glutamine levels should be tested as an adjunctive therapeutic approach. It is also clear that glutamine facilitates host-protective M1 polarization of the infected mouse macrophages, enhancing their antimycobacterial and inflammatory properties (65). In HIV patients, glutamine supplementation (20 g/day for 7 days) has improved serum glutamine levels (68).

While glutamine supplementation may represent a promising approach, the *ex-vivo* macrophage infection model is unsuitable for these studies as the macrophage culture media already contains milli-molar concentrations of glutamine. Testing glutamine supplementation in murine models of TB as an adjunctive therapy, despite being non-trivial, holds promise.

## Glutamine metabolism inhibition as a host-directed therapy for TB

Several strategies have been employed to target glutamine metabolism in cancer models. These strategies can be majorly classified into four categories; (1) glutaminase inhibitors, (2) glutamine transporter inhibitors, (3) glutamine synthetases inhibitors, and (4) pleiotropic glutamine antagonists (Table 1, Figure 1) (98–100). While numerous studies have tested these approaches in various cancer models, only a handful of such studies have been performed in experimental models of TB. In a study by Koekan et al. (63) pharmacological inhibitors such as GPNA, BPTES, C968, and DON were shown to adversely impact the cytokine production by *Mtb*-stimulated peripheral blood mononuclear cells, particularly T-cell cytokines such as IFNy, IL-22, and IL-17. However, the impact of these inhibitors on the bacillary burden was not explored. More recently, Roca et al. (69) have demonstrated that TNF-treated infected macrophages upregulate glutaminolysis to induce pathogenic mitochondrial reactive oxygen species production (mROS). The pathogenic mROS production in macrophages could be successfully perturbed by BPTES and CB-839 (GLS1 inhibitors) and R162 (GLUD1 inhibitor) (69). Interestingly, the effect of GLS1 inhibitors was limited to the infected macrophages with excess TNF. A more recent report has shown that the glutamine metabolism antagonist prodrug, JHU083, significantly reduced the lung bacillary burden, improving lung histopathology and survival of *Mtb-*infected mice. The authors also noted significant immunological and metabolic reprogramming in the infected lungs, potentially driving the therapeutic benefit. As the drug was administered early after infection, future studies in acute and chronic models of TB infections will be required to assess the true translational potential of the drug (66, 67). All these early studies suggest that glutamine metabolism inhibition has the potential to be developed as an effective host-directed therapy option for TB.

**Table 1:** List of glutamine metabolism inhibitors used in various pathological conditions.

**Drug**	**Host target**	**Condition**	**Reference**
CB-839 (Telaglenastat)	GLS1	Melanoma, renal cell carcinoma, NSCLC, CRC, head & neck squamous carcinoma, *M. marinum*/Zebrafish	(69, 70)
C968	Glutaminase C	Ovarian cancer, NSCLC, breast cancer, *Mtb-*stimulated PBMCs	(63, 71, 72)
Epigallocatechin gallate (R162)	GLUD1 and GLUD2	Neuroblastoma, glioma, and CRC cells	(73)
Bis-2-(5-phenylacetamido-1,2,4-thiadiazol-2-yl) ethyl (BPTES)	GLS1 but not GLS2	Aspergillosis, lymphomas, breast cancer, CRC and, ovarian cancers, *M. marinum*/zebrafish	(56, 74)
Purpurin analog R162	GLUD1	Breast, NSCLC, and glioma cancer, *M. marinum*/Zebrafish	(75, 76)
Sulfasalazine	SLC7A11	Triple-negative breast cancer (TNBC)	(77)
Erastin	SLC7A11	Pancreatic ductal adenocarcinoma	(78, 79)
Imidaole Ketone Erastin, and Peperazine Erastin	SLC7A11	Large B-cell lymphoma and fibrosarcoma	(80, 81)
Sorafenib	SLC7A11	Hepatocellular carcinoma	(82, 83)
Benzylserine	SLC1A5 (non-specific)	Breast cancer	(84)
Benzylcysteine	SLC1A5 (non-specific)	Gastric cancer	(85)
L-γ-Glutamyl-p-nitroanilide (GPNA)	SLC1A5 (non-specific)	TNBC, Lung and neuroblastoma cancers, aspergillosis, *M. marinum*/zebrafish	(56, 86–88)
Synthetic Monoclonal Antibodies (KM4008, KM4012, and KM4018)	SLC1A5	CRC	(89)
V-9302	SLC1A5, (non-specific binding to SLC38A2 & SLC7A5)	Lung cancer, oral mucosa carcinomas, osteoporosis	(90, 91)
N-methyl-aminoisobutyric acid (MeAIB)	SLC38A1 and/or SLC38A2	Cervical and osteosarcoma cancer cells	(92, 93)
6-diazo-5-oxo-norleucine (DON)	Glutamine antagonist	Aspergillosis, cancer, TB	(56, 66, 94)
Sirpiglenastat (DRP-104) and its analog (JHU083)	Glutamine antagonist Prodrug	Breast, pancreatic and renal cancers, TB, EcoHIV	(39, 40, 95)
PF-04859989	GOT1 (Glutamic oxaloacetic transaminase 1)	Pancreatic ductal adenocarcinoma	(96)
Aspulvinone O	GOT1	Pancreatic ductal adenocarcinoma	(97)

M. marinum, Mycobacterium marinum; C968, 5-(3-bromo-4-(dimethylamino) phenyl)-2,2-dimethyl-2,3,5,6-tetrahydrobenzo[a]phenanthridin-4(1H)-one; NSCLC, non-small-cell Lung cancer; CRC, colorectal cancer; PBMCs, peripheral blood mononuclear cells.

## Future of glutamine metabolism in TB pathogenesis

More studies are required to demonstrate the precise link between glutamine metabolism and TB pathogenesis. Several research avenues need further investigation. First and foremost, what signals/factors trigger the upregulation of glutamine metabolism during *Mtb* infection? Are these signals/triggers host-derived or secreted by *Mtb*? Is glutamine metabolism upregulation an early event during *Mtb* infection or a sustained phenotype of infected lungs? Does glutamine metabolism upregulation contribute to granuloma formation? And if yes, how does glutamine metabolism upregulation contribute to granuloma formation? How, why, and when do granulomas undergo metabolic reprogramming during different phases of infection? What are the differences in the metabolic profile of granuloma vs. non-granulomatous regions of *Mtb*-infected lungs? Do all lung cells undergo similar metabolic reprogramming, or is the effect driven by a handful of cell types? Do non-immune lung cells also undergo metabolic reprogramming upon *Mtb* infection? While numerous questions can be asked in the context of glutamine metabolism and TB pathogenesis, a solid first step could be investigating the impact of *Mtb* upon the metabolic profiles of the immune cells and using the knowledge to block glutamine metabolism mindfully and selectively. One of the important considerations will be to strike a delicate balance of selective and targeted inhibition of the problematic immunosuppressive cell populations while maintaining the wellbeing of host-protective cell types (38).

## Challenges of the new field and potential solutions

Investigating the impact of glutamine metabolism on TB pathogenesis will require extensive deployment of metabolomics tools. There are a few unique challenges specific to TB research: first and foremost, as single-cell metabolomics is still in the initial phases of development, metabolomics of individual cell types remains challenging. Worldwide, most TB researchers (including those in developed countries) do not have access to a flow sorter in a BSL3-containment facility, making it harder to work with individual cell types. While immortalized immune cell lines are an option, magnetic bead-based enrichment of the primary lung-derived cells may be helpful in several cases. Over the years, there has been tremendous development and expansion of bead-based kits. However, these kits need extensive standardization and optimization when working with complex tissue samples. The second challenge will be distinguishing the host vs. bacterial-derived metabolites in the infected samples. Novel isotope labeling methods may need to be developed to circumvent this issue. Third, metabolite fluxes tend to be extremely sensitive to their milieu and are potentially affected during tissue collection, processing, sorting, and metabolite extraction, causing extensive batch-to-batch variations and processing artifacts. Fast, efficient, and homogenous processing of the infected samples, while not impossible to achieve, all these factors can be challenging in a BSL3 containment facility. Such processing artifacts can be avoided using stable isotope-labeled nutrients (e.g., [^13^C] glucose or [^13^C] glutamine) to probe the metabolic fluxes within the intact lungs *in vivo*. This method was recently developed by Faubert et al. (101) and was used to investigate the metabolic activity of tumors *in vivo* using the [^13^C] glucose label. Despite the unique challenges faced by TB researchers, the glutamine metabolism and TB pathogenesis field holds tremendous promise and needs our immediate attention.

## Conclusion

In conclusion, sufficient data supports the claim that glutamine metabolism is a promising host-directed mechanism that can be targeted to perturb TB pathogenesis. Several inhibitors, originally identified by cancer researchers, can be tested as potential host-directed therapy. However, several missing pieces in the puzzles must be placed to successfully predict and implement these therapies and evaluate their therapeutic potential.

## Author contributions

SP: Conceptualization, Funding acquisition, Investigation, Software, Supervision, Visualization, Writing—original draft, Writing—review & editing. WRB: Conceptualization, Funding acquisition, Software, Supervision, Visualization, Writing—review & editing.
